# Supplementary material for: Hydrothermal Synthesis of Aqueous-Soluble Copper Indium Sulfide Nanocrystals and Their Use in Quantum Dot Sensitized Solar Cells
Source: Nanomaterials (Basel). 2020 Jun 28;10(7):1252. doi: 10.3390/nano10071252 (PMC7407332; doi:10.3390/nano10071252)
Supplement: Supplementary file 1 [file nanomaterials-10-01252-s001.pdf]

nanomaterials

MDPI

Supplementary Materials

Hydrothermal Synthesis of Aqueous-Soluble Copper Indium Sulfide Nanocrystals and Their Use in Quantum Dot Sensitized Solar Cells

Calink I. L. Santos [1,2], Wagner S. Machado [1,2], Karl David Wegner [2], Leiriana A. P. Gontijo [1], Jefferson Bettini [3], Marco A. Schiavon [1], Peter Reiss [2] and Dmitry Aldakov [2,*]

[1] Grupo de Pesquisa em Química de Materiais (GPQM), Departamento de Ciências Naturais, Universidade Federal de São João del-Rei, Campus Dom Bosco, Praça Dom Helvécio, 74, CEP 36301-160, São João del-Rei, MG, Brazil; calinkindiaradc@hotmail.com (C.I.L.S.); wagner@ufsj.edu.br (W.S.M.); leiriana@hotmail.com (L.A.P.G.); schiavon@ufsj.edu.br (M.A.S.)

[2] Univ. Grenoble Alpes, CEA, CNRS, IRIG-SyMMES, STEP, 38000 Grenoble, France; karl-david.wegner@bam.de (K.D.W.); peter.reiss@cea.fr (P.R.)

[3] Laboratório Nacional de Nanotecnologia, Centro Nacional de Pesquisa em Energia e Materiais, CEP 13083-970, Campinas–SP, Brazil; jefferson.bettini@lnnano.cnpem.br

* Correspondence: dmitry.aldakov@cea.fr

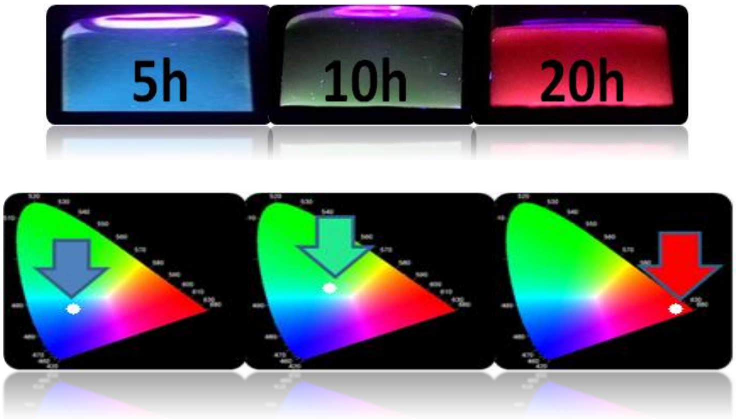

Figure S1. Sample photographs (top) and respective chromatic diagrams (bottom).

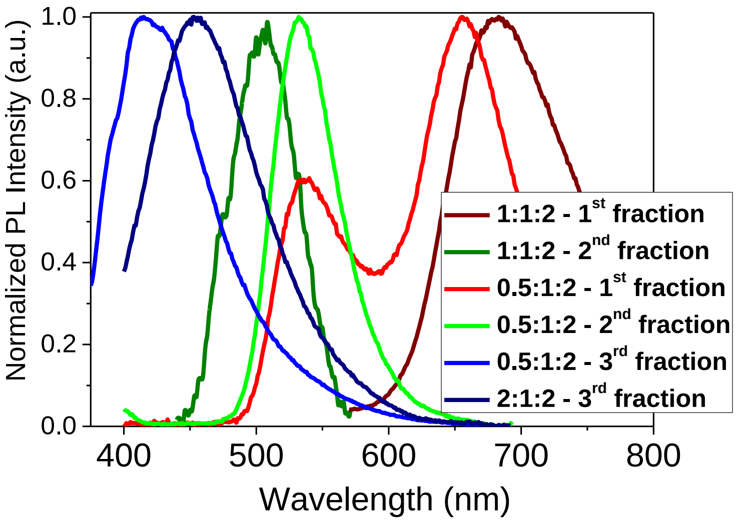

Figure S2. Influence of the precursors ratio on CIS QD emission properties.

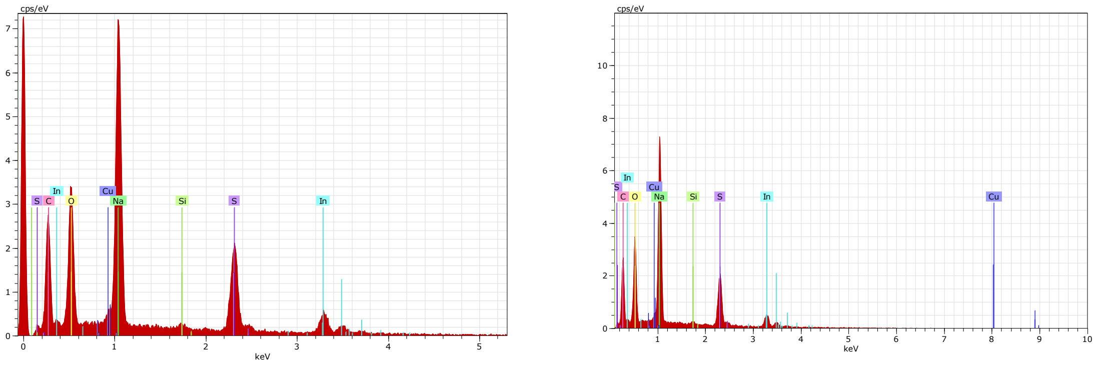

(**a**) (**b**)

Figure S3. Energy dispersive spectra (EDX) for CIS-1 (**a**) and CIS-2 (**b**).

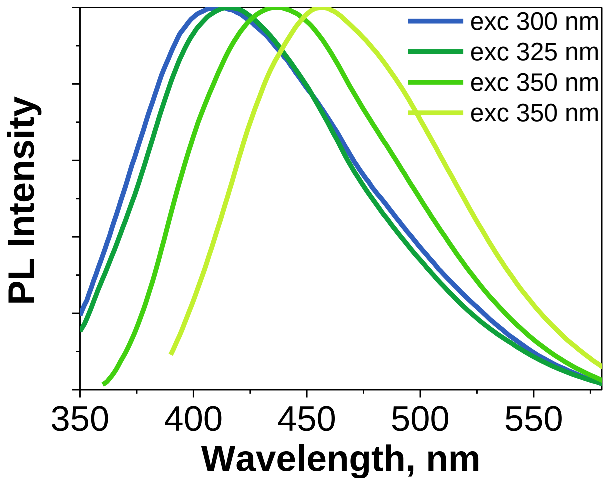

Figure S4. Excitation wavelength dependence of the PL emission spectra of the 3rd fraction CIS-3.

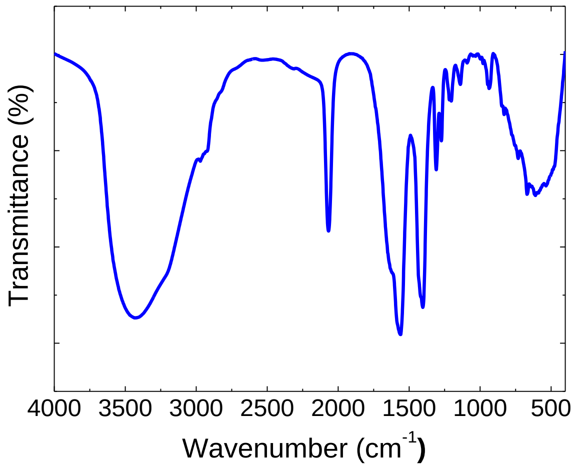

Figure S5. IR spectrum of CIS-3. A band at around 2085 cm^{-1} related to the presence of isothiocyanate is distinguishable, which possibly comes from the unreacted thiourea.

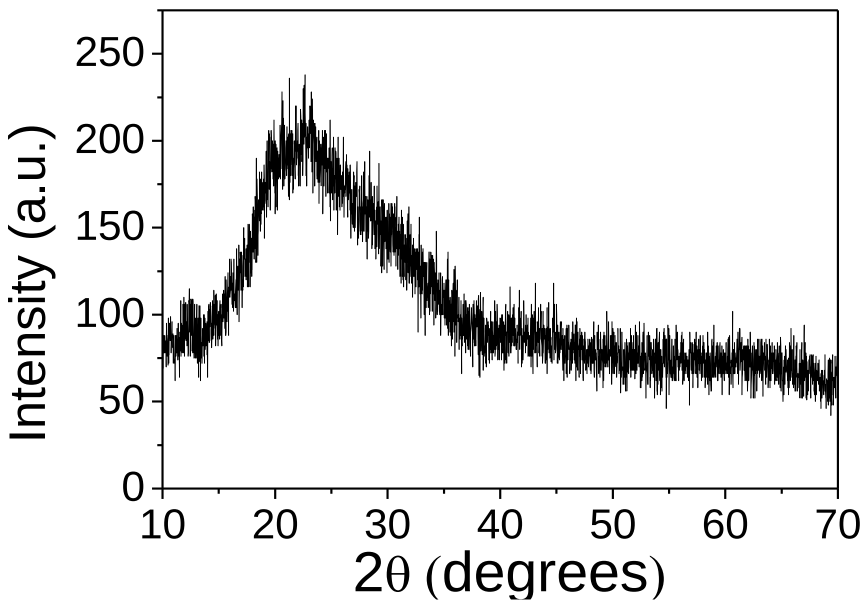

Figure S6. X-ray diffractogram of the CIS-3.

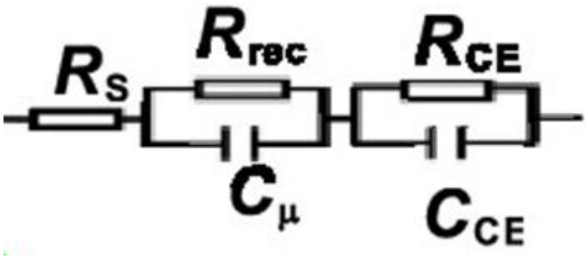

Scheme S1. Standard model used for fitting the Nyquist plots with Nova software according to a standard fitting model [1].

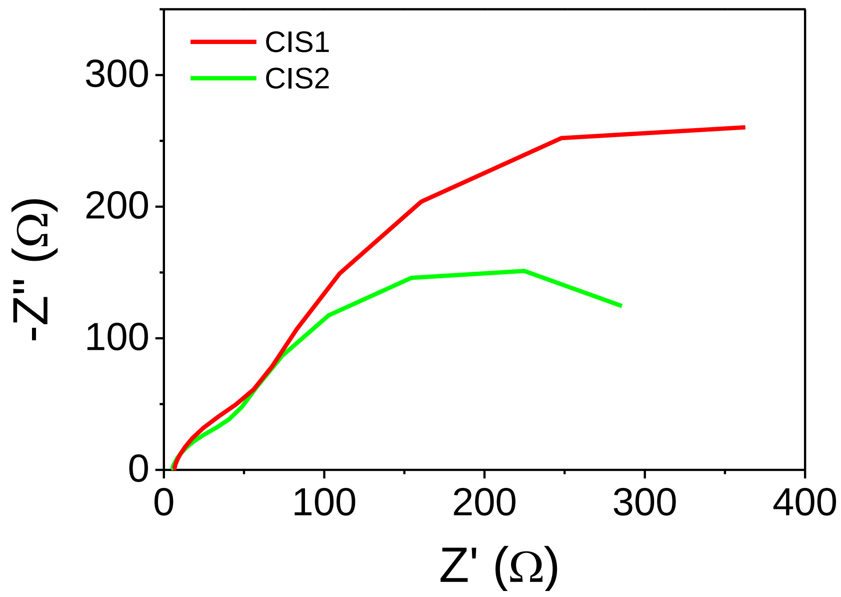

Figure S7. Nyquist plot of QDSSCs using CIS fractions as sensitizers at applied potential of 0.55 V.

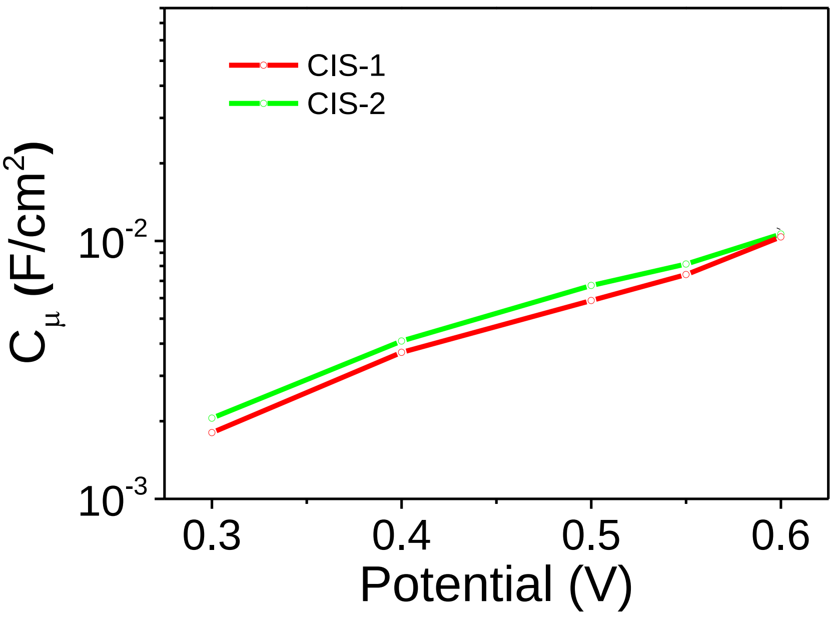

Figure S8. Chemical capacitance of QDSSCs using CIS fractions as a function of bias potential.

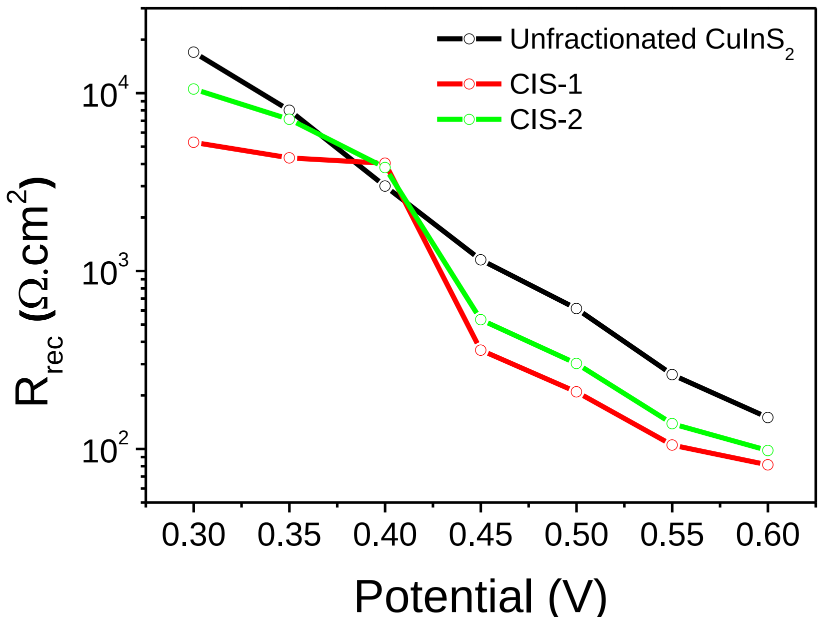

Figure S1. Recombination resistance for several CIS QDSSC devices as a function of applied potential

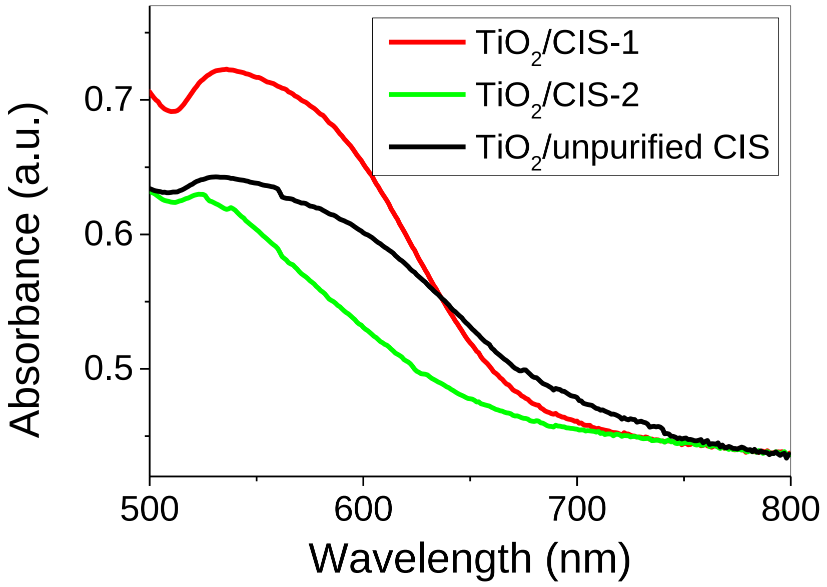

Figure S10. Absorption spectra of the TiO_2 electrodes sensitized with fractionated and non-fractionated $CuInS_2$ QDs.

ZnO nanowires synthesis. ZnO nanowires were directly grown on fluorine-doped tin oxide-coated (FTO) substrates (Figure S11) by electrochemical deposition in aqueous medium. For this, 0.01 M solutions of hexamethylenetetramine and zinc nitrate were prepared. About 80 mL were used for deposition. After careful cleaning of the substrates, electrochemical deposition was carried out in an electrochemical cell, using Pt wire as a counter-electrode, Ag/AgCl as reference, and FTO substrate as working electrode. The system was then heated to 90 °C, and a potential of −1 V was applied for 30 min. After the synthesis, the samples were annealed at 300°C for 1 h.

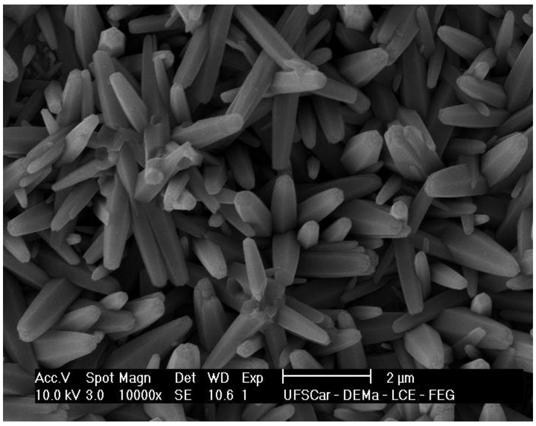

Figure S11. SEM image of ZnO nanowires grown on fluorine-doped tin oxide-coated (FTO) substrates.

Table S1. Reported hydrothermal syntheses of $CuInS_2$ QDs.

QDs	Ligand*	Synthesis temperature	Size, nm	Quantum yield	Ref
$CuInS_2$	MPA	150°	2–4	3.3%	[2]
$CuInS_2$/ZnS	TGA	116°	3–5	40%	[3]
$CuInS_2$	L-cys	150°	-	-	[4]
CuInZnS/ZnS	GSH	150°	2–3	35%	[5]
CuInZnS/ZnS	GSH + PSSA-co-MA	200°	4	27%	[6]
$CuInS_2$/ZnS	L-cys	100°	3–5	0.1%	[7]
CuInZnS	MPA	120–180°	4–8	11.3%	[8]
$CuInS_2$	MPA	150°	2–4	1–9%	This work

* MPA: 3-mercaptopropionic acid, TGA: thioglycolic acid, L-cys: L-cysteine, GSH: glutathione, PSSA-co-MA: poly (styrenesulfonic acid-co maleic acid).

Table S2. Solar cells using $CuInS_2$ QDs synthesized in water as sensitizers.

Ligand	Synthesis conditions	V_{OC} (V)	J_{SC} (mA/cm^2)	FF	η (%)	Ref
TGA	Heating up	0.60	9.6	42	8.15[a]	[9]
TAA[b]	Heating up	0.90	14.2	49	6.33	[10]
TGA	Microwave	0.91	14.0	48	6.12	[11]
CYS	Microwave	0.65	19.2	47	5.90	[12]
TGA	Heating up	0.58	19.3	48	5.38	[13]
MPA	Hydrothermal	0.52	14.0	64	4.67	This work
TGA	Heating up[c]	0.86	9.8	52	4.39	[14]
TGA	Heating up	0.75	8.7	53	3.54	[15]
TAAm[d]	Hydrothermal	0.73	4.7	58	2.09	[16]
TGA	Heating up	0.49	8.1	37	1.47	[17]

[a] Light intensity: 0.3 Sun; [b] TAA: thioacetic acid; [c] Mn-doped QDs; [d] TAAm: thioacetamide.

Table S3. Ratios between the charges of QD core-forming ions ([Cu]+[In]*3)/([S]*2).

Ratio	CIS-1	CIS-2
**Q_{cat}/Q_{an}**	0.98	0.31

Table S4. Zeta potential measurements data.

Sample	Zeta Potential (mV)	Mobility (cm^2/Vs)	Conductivity (mS/cm)
CIS-1	-38.12	1.86×10^{-4}	11.34
CIS-2	-45.00	2.20×10^{-4}	2.85

CIS-3	-50.83	2.48×10^{-4}	0.32

Table S5. Photovoltaic parameters of TiO_2 based solar cells sensitized by CIS NCs using CuS counter-electrodes.

Fraction	V_{OC} (V)	J_{SC} (mA/cm^2)	FF	η (%)
NF	0.55	11.7	0.42	2.69
CIS-1	0.53	7.9	0.50	2.10
CIS-2	0.52	7.6	0.49	1.94

Table S6. Stability of the QDSSCs based on unpurified $CuInS_2$ QDs using CuS counter-electrode.

Days after fabrication	V_{OC} (V)	J_{SC} (mA/cm^2)	FF	η (%)
0	0.55	11.65	0.42	2.69
1	0.55	11.70	0.42	2.67
21	0.57	11.20	0.50	3.19

Table S7. Electrical properties of the TiO_2/CIS cells as measured by EIS from the literature and those obtained in this work.

QD synth.	CE	V_{appl} (V)	R_S (Ω.cm^2)	R_{CE} (Ω.cm^2)	R_{rec} (Ω.cm^2)	C_μ (mF.cm^{-2})	τ_n (ms)	Ref
Org	Cu_2S/Brass	0.55	5.48	2.09	30.1	21.74	654	[18]
Org	Cu_2S/Brass	0.55	15	--	73.1	5.04	438	[19]
Org	Cu_2S/Brass	0.60	17.33	5.94	258.6	3.95	1022	[20]
Aq	Cu_2S/Brass	0.60	1.7	0.22	67	11	737	This work
Aq	CuS/FTO	0.55	1.8	0.29	222	7.9	1778	

Table S8. Photovoltaic performance of the QDSSCs based on ZnO nanowires.

Sample	V_{OC} (V)	J_{SC} (mA/cm^2)	FF (%)	η (%)
**CIS-1**	0.10	0.59	29	0.017
**CIS-2**	0.05	0.12	20	0.001
**CIS nf**	0.03	4.03	27	0.032

References

1. Bisquert, J.; Fabregat-Santiago, F.; Mora-Seró, I.; Garcia-Belmonte, G.; Giménez, S. Electron lifetime in dye-sensitized solar cells: Theory and interpretation of measurements. *J. Phys. Chem. C* **2009**, *113*, 17278–17290, doi:10.1021/jp9037649.
2. Liu, S.; Zhang, H.; Qiao, Y.; Su, X. One-pot synthesis of ternary $CuInS_2$ quantum dots with near-infrared fluorescence in aqueous solution. *RSC Adv.* **2012**, *2*, 819, doi:10.1039/c1ra00802a.
3. Chen, Y.; Li, S.; Huang, L.; Pan, D. Low-cost and gram-scale synthesis of water-soluble Cu-In-S/ZnS core/shell quantum dots in an electric pressure cooker. *Nanoscale* **2014**, *6*, 1295–1298, doi:10.1039/c3nr05014a.
4. Gao, X.; Liu, Z.; Lin, Z.; Su, X. $CuInS_2$ quantum dots/poly((L)-glutamic acid)-drug conjugates for drug delivery and cell imaging. *Analyst* **2014**, *139*, 831–836, doi:10.1039/c3an01134h.
5. Jiang, T.; Song, J.; Wang, H.; Ye, X.; Wang, H.; Zhang, W.; Yang, M.; Xia, R.; Zhu, L.; Xu, X. Aqueous synthesis of color tunable Cu doped Zn–In–S/ZnS nanoparticles in the whole visible region for cellular imaging. *J. Mater. Chem. B* **2015**, *3*, 2402–2410, doi:10.1039/C4TB01957A.
6. Vinayagam, J.; Chen, G.R.; Huang, T.Y.; Ho, J.H.; Ling, Y.C.; Ou, K.L.; Chang, J.Y. Aqueous synthesis of CuInZnS/ZnS quantum dots by using dual stabilizers: A targeting nanoprobe for cell imaging. *Mater. Lett.* **2016**, *173*, 242–247, doi:10.1016/j.matlet.2016.03.041.
7. Spangler, L.C.; Chu, R.; Lu, L.; Kiely, C.J.; Berger, B.W.; McIntosh, S. Enzymatic biomineralization of biocompatible $CuInS_2$, $(CuInZn)S_2$ and $CuInS_2$/ZnS core/shell nanocrystals for bioimaging. *Nanoscale* **2017**, *9*, 9340–9351, doi:10.1039/C7NR02852K.

8. Chen, X.; Chen, S.; Xia, T.; Su, X.; Ma, Q. Aqueous synthesis of high quality multicolor Cu-Zn-In-S quantum dots. *J. Lumin.* **2017**, *188*, 162–167, doi:10.1016/j.jlumin.2017.04.019.
9. Raevskaya, A.; Rosovik, O.; Kozytskiy, A.; Stroyuk, O.; Dzhagan, V.; Zahn, D.R.T.T. Non-stoichiometric Cu–In–S@ZnS nanoparticles produced in aqueous solutions as light harvesters for liquid-junction photoelectrochemical solar cells. *RSC Adv.* **2016**, *6*, 100145–100157, doi:10.1039/C6RA18313A.
10. Higashimoto, S.; Nakase, T.; Mukai, S.; Takahashi, M. Copper-indium-sulfide colloids on quantum dot sensitized TiO_2 solar cell: Effects of capping with mercapto-acid linker molecules. *J. Colloid Interface Sci.* **2019**, *535*, 176–181, doi:10.1016/j.jcis.2018.09.092.
11. Higashimoto, S.; Murano, M.; Arase, T.; Mukai, S.; Azuma, M.; Takahashi, M. Highly qualified copper-indium sulfide colloids prepared in water under microwave irradiation and their applications to the TiO_2 based quantum dot-sensitized solar cells. *Sol. Energy Mater. Sol. Cells* **2017**, *169*, 203–209, doi:10.1016/j.solmat.2017.05.005.
12. Chang, J.Y.; Li, C.H.; Chiang, Y.H.; Chen, C.H.; Li, P.N. Toward the facile and ecofriendly fabrication of quantum dot-sensitized solar cells via thiol coadsorbent assistance. *ACS Appl. Mater. Interfaces* **2016**, *8*, 18878–18890, doi:10.1021/acsami.6b05411.
13. Luo, J.; Wei, H.; Huang, Q.; Hu, X.; Zhao, H.; Yu, R.; Li, D.; Luo, Y.; Meng, Q. Highly efficient core-shell $CuInS_2$-Mn doped CdS quantum dot sensitized solar cells. *Chem. Commun. (Camb).* **2013**, *49*, 3881–3883, doi:10.1039/c3cc40715b.
14. Higashimoto, S.; Okada, T.; Arase, T.; Azuma, M.; Yamamoto, M.; Takahashi, M. High performance of TiO_2 based solar cells sensitized with copper-indium sulfide colloids prepared in water: Roles of surface modifications with indium sulfide and zinc sulfide by SILAR methods. *Electrochim. Acta* **2016**, *222*, 867–874, doi:10.1016/j.electacta.2016.11.051.
15. Higashimoto, S.; Inui, S.; Nakase, T.; Azuma, M.; Yamamoto, M.; Takahashi, M. Inorganic dye-sensitized solar cell employing In-enriched Cu-In-S ternary colloids prepared in water media. *RSC Adv.* **2015**, *5*, 71743–71748, doi:10.1039/c5ra10751b.
16. Mousavi-Kamazani, M.; Salehi, Z.; Motevalli, K. Enhancement of quantum dot-sensitized solar cells performance using $CuInS_2$–Cu_2S nanocomposite synthesized by a green method. *Appl. Phys. A Mater. Sci. Process.* **2017**, *123*, 1–8, doi:10.1007/s00339-017-1325-3.
17. Hu, X.; Zhang, Q.; Huang, X.; Li, D.; Luo, Y.; Meng, Q. Aqueous colloidal $CuInS_2$ for quantum dot sensitized solar cells. *J. Mater. Chem.* **2011**, *21*, 15903, doi:10.1039/c1jm12629f.
18. Pan, Z.; Mora-Sero, I.; Shen, Q.; Zhang, H.; Li, Y.; Zhao, K.; Wang, J.; Zhong, X.; Bisquert, J.; Mora-Seró, I.; et al. High-efficiency "green" quantum dot solar cells. *J. Am. Chem. Soc.* **2014**, *136*, 9203–9210, doi:10.1021/ja504310w.
19. Wang, G.; Wei, H.; Shi, J.; Xu, Y.; Wu, H.; Luo, Y.; Li, D.; Meng, Q. Significantly enhanced energy conversion efficiency of $CuInS_2$ quantum dot sensitized solar cells by controlling surface defects. *Nano Energy* **2017**, *35*, 17–25, doi:10.1016/j.nanoen.2017.03.008.
20. Yue, L.; Rao, H.; Du, J.; Pan, Z.; Yu, J.; Zhong, X. Comparative advantages of Zn–Cu–In–S alloy QDs in the construction of quantum dot-sensitized solar cells. *RSC Adv.* **2018**, *8*, 3637–3645, doi:10.1039/C7RA12321C.
